# Copper and Zinc Feud: Is This Myelodysplasia or Myelodysplastic Syndrome?

**DOI:** 10.7759/cureus.26789

**Published:** 2022-07-12

**Authors:** Manoj Kumar, Sushmitha Nanja Reddy, Rana Ismail

**Affiliations:** 1 Hematology and Oncology, Ascension Providence Hospital, Southfield, USA; 2 Internal Medicine, Hennepin County Medical Center, Minneapolis, USA; 3 Internal Medicine, Detroit Medical Center Sinai Grace Hospital / Wayne State University, Detroit, USA; 4 College of Osteopathic Medicine, Michigan State University, Detroit, USA

**Keywords:** sideroblastic anemia, pancytopenia, zinc, myelodysplastic syndrome, copper deficiency

## Abstract

We report a case of a 59-year-old male who developed pancytopenia and multiorgan failure attributed to copper deficiency from exogenous consumption of zinc tablets. During the six months preceding his presentation, he had experienced increasing shortness of breath, lightheadedness, and fatigue. Laboratory studies revealed pancytopenia with profound anemia (hemoglobin level 2.8 g/dL) along with evidence of acute kidney injury and acute heart failure; the patient was presumed to have multiorgan failure due to profound anemia. Bone marrow biopsy revealed dyspoiesis suggestive of myelodysplastic syndrome (MDS). There were no cytogenetic abnormalities observed. However, the blood workup analysis found low copper and ceruloplasmin levels, whereas zinc levels were excessively elevated (257 mg/dL). Upon inquiry, the patient reported taking an over-the-counter zinc supplement of an unknown quantity for over a year. After two months of copper treatment, his blood count returned to normal. This case highlights a rare presentation of zinc-induced copper deficiency resulting in pancytopenia and severe anemia-related multiorgan failure. A growing number of hematological disorders are being linked to copper deficiency. Copper deficiency pancytopenia is a reversible condition that often goes unnoticed and can be misdiagnosed as MDS because it has similar hematological characteristics.

## Introduction

Copper deficiency is a relatively rare condition because the recommended dietary requirement for copper is only 900 mcg per day, typically obtained through a regular diet unless other predisposing factors are present. Normal serum copper levels range from 70 to 125 mcg per day [1]. Copper deficiency can induce pancytopenia and dysplastic hematopoiesis, therefore, mimicking myelodysplastic syndrome (MDS). However, in contrast to MDS, copper deficiency does not result in cytogenetic abnormalities on the karyotype. The most prominent bone marrow morphologic features include ringed sideroblasts, intracytoplasmic vacuoles in erythroblasts, and granulocyte precursors. According to studies, hematogone hyperplasia with mature B lineage precursors can be found by flow cytometry in copper deficiency-related dysplasias, whereas these are missing or infrequent in MDS, which is defined by a deficiency in B cell progenitors as shown by DNA microarray analyses [2]. Due to the competition between zinc and copper for intestinal absorption, increased zinc levels may result in copper deficiency or insufficiency.

## Case presentation

A 59-year-old man with a history of obstructive sleep apnea presented to the hospital with progressively worsening shortness of breath, lightheadedness, and fatigue over six months. His social history was notable for his poor diet, with most of his meals consisting of TV dinners and canned goods. Examination revealed that he had conjunctival pallor. The results of the laboratory tests were as follows: hemoglobin level 2.8 g/dL, mean corpuscular volume (MCV) 110.5 fL, white blood cell (WBC) count 2.0 k/cu.mm, absolute neutrophil count 0.6 k/cu.mm, lactic acid 12.9 mmol/L, serum creatinine 2.28 mg/dL, NTproBNP 1492 pg/mL, and troponin I 1.229 mg/dL. He was presumed to have a multiorgan failure with acute renal damage and heart failure due to his profound anemia. Bone marrow biopsy revealed dyspoiesis predominantly characterized by vacuoles in erythroid and myeloid precursors and numerous ringed sideroblasts suggesting MDS. There were no cytogenetic abnormalities observed. Serum B12 level was normal at 387 pg/dL. His symptoms improved after transfusion, and he remained dependent on transfusions and granulocyte stimulating factors for anemia and neutropenia during his follow-up appointments at the hematology clinic. After investigating his pancytopenia and abnormal bone marrow results, his ceruloplasmin and copper levels were found to be exceedingly low at 3.0 and 7 mg/dL, respectively, but his serum zinc level was elevated at 257 mg/dL. Further investigation revealed that the patient had consumed unknown quantities of over-the-counter zinc tablets for more than a year. He was advised to stop taking zinc and start taking copper supplements. As a result, his blood count levels improved substantially and reverted to normal within two months; he no longer requires granulocyte stimulating factors or blood transfusions.

## Discussion

Myelodysplastic syndrome is a set of clonal hematopoietic stem cell disorders marked by cytopenias, ineffective hematopoiesis, and a higher risk of progression to acute myeloid leukemia. There are several reversible hematologic abnormalities similar to MDS, such as human immunodeficiency virus infection-induced cytopenia, medications (valproic acid, ganciclovir, alemtuzumab, mycophenolate mofetil, methotrexate), excessive alcohol consumption, and dietary deficiencies such as folate, vitamin B12, and copper insufficiency.

Copper deficiency produces a spectrum of hematological disorders, including microcytic or normocytic anemia, sideroblastic anemia, leukopenia, or pancytopenia with blasts mimicking MDS. Copper deficiency is often caused by malabsorption due to gastric bypass surgery, small intestinal disorders, cystic fibrosis, severe malnutrition, total parenteral nutrition, and zinc toxicity. Metals like copper and zinc are absorbed into the mucosal cells by attaching to intracellular ligands like metallothionein. Interestingly, a high oral zinc consumption stimulates metallothionein synthesis in the intestinal cells. However, zinc and copper compete for intestinal absorption, but copper has a stronger affinity for metallothionein than zinc; therefore, a high zinc intake hinders copper absorption [3]. Consequently, a sustained high zinc consumption can result in a severe copper deficit.

Copper is essential to many transport proteins, including hephaestin and ceruloplasmin ferroxidase. Hephaestin facilitates iron absorption from intestinal cells, whereas ceruloplasmin ferroxidase, a major copper-carrying protein in the blood, is involved in iron metabolism. Hypochromia and microcytosis are likely caused by copper deficiency due to the role of ceruloplasmin ferroxidase in iron metabolism. Mitochondrial cytochrome oxidase fails to synthesize heme from ferric iron and protoporphyrin at the average rate, possibly resulting in ringed sideroblasts, also known as mitochondrial iron accumulation [4]. Copper is a crucial cofactor for some enzymes involved in cell division and protein synthesis, and thus its deficiency may result in neutropenia and macrocytosis.

Copper is a necessary cofactor for some enzymes involved in cell division and protein synthesis; hence, copper deficiency can lead to neutropenia and macrocytosis. However, little is known about how copper deficiency causes MDS-like myeloid changes. In certain studies, copper deficiency has been demonstrated to slow hematopoietic progenitor cell differentiation and self-renewal, which could cause MDS-like symptoms [5]. In patients with MDS, mutations in cytochrome c-oxidase, the terminal complex of the mitochondrial respiratory chain, are more prevalent or are observed at a higher frequency [6]. The catalytic core of cytochrome c-oxidase contains two copper-containing subunits, and copper deficiency has been linked to cytochrome c-oxidase dysfunction in the cardiac muscles of rats [7]. It is likely that such dysfunction in cellular oxidation also contributes to poor hematopoiesis, a process with a high cellular turnover rate and high energy consumption. Other possibilities include the generation of anti-neutrophil antibodies in neutropenic patients with copper deficiency, myeloid dyspoiesis, shorter cellular life spans caused by increased oxidative stress and decreased superoxide dismutase activity, and more [8].

## Conclusions

Screening for copper deficiency in patients with cytopenias is still not a common practice. Many cases are diagnosed with cytopenia, which could sometimes be misdiagnosed as MDS. Therefore, copper levels should be strongly considered when cytopenias and bone marrow morphologic abnormalities are investigated for MDS in the absence of a clonal cytogenetic aberration. Pancytopenia induced by copper deficiency is quickly reversible with oral copper preparations containing at least 2 mg of elemental copper per day, and normal counts can be restored in less than two months.
